# Ten tips for the first hemodialysis sessions

**DOI:** 10.1093/ckj/sfag270

**Published:** 2026-08-12

**Authors:** Manfred Hecking, Michael Kolland, Marieta Theodorakopoulou, Janosch Niknam, Sebastian Mussnig, Dilara Gülmez, Alexander Kirsch, Gaetano Alfano, Christian Combe, Rumeyza Kazancioglu, Björn Meijers, Casper Franssen, Yuri Battaglia

**Affiliations:** Department of Medicine III, Division of Nephrology and Dialysis, Medical University of Vienna, Vienna, Austria; Department of Internal Medicine, Clinical Division of Nephrology, Medical University of Graz,, Graz, Austria; First Department of Nephrology, Hippokration Hospital, Aristotle University of Thessaloniki, Thessaloniki, Greece; Department of Medicine III, Division of Nephrology and Dialysis, Medical University of Vienna, Vienna, Austria; Department of Medicine III, Division of Nephrology and Dialysis, Medical University of Vienna, Vienna, Austria; Department of Medicine III, Division of Nephrology and Dialysis, Medical University of Vienna, Vienna, Austria; Department of Internal Medicine, Clinical Division of Nephrology, Medical University of Graz,, Graz, Austria; Nephrology Dialysis and Kidney Transplant Unit, Azienda Ospedaliero Universitaria di Modena, Modena, Italy; Bordeaux School of Public Health, Université de Bordeaux, INSERM U1219, CIC 1401, Bordeaux, France; Department of Nephrology and Nutrition, Hospices Civils de Lyon, Centre Hospitalier Lyon-Sud, Pierre-Bénite, France; Centre Hospitalier d’Ardèche Méridionale, Aubenas, France; Division of Nephrology, School of Medicine, Bezmialem Vakif University, Istanbul, Türkiye; Nephrology and Renal Transplantation Research Group, Department of Microbiology, Immunology and Transplantation, KU Leuven, Leuven, Belgium; Department of Nephrology, UZ Leuven, Leuven, Belgium; University Medical Center Groningen, Department of Nephrology, University of Groningen, Groningen, The Netherlands; Center for Medical Science, University of Trento,, Trento, Italy; Nephrology and Dialysis Unit, Santa Chiara Hospital, ASUIT, Trento, Italy

**Keywords:** chronic renal failure, dialysis, dry weight, hemodialysis, hemodiafiltration

## Abstract

The start of hemodialysis is a particularly vulnerable phase in a patient’s journey through various stages of kidney disease: Early mortality is high, functional decline is common, and many patients feel poorly informed and unprepared for what dialysis entails. Decisions made during this phase shape long-term trajectories. Here, we aim at providing “suggestions for colleagues” rather than guideline-like recommendations. In our six tips regarding dialysis consent, transplant candidacy, pill check, vaccination, exercise, and nutrition, we cover the direct patient–doctor axis. In our four tips on volume management, modality, anticoagulation, and incremental hemodialysis, we provide advice that may benefit patients through the prescription itself. Our choice of items is incomplete, as important aspects like vascular access, anemia management, mineral bone disorder, patient-reported symptoms, and dialysate composition are missing. Altogether, we hope that our contribution can emphasize, that hemodialysis may be viewed as the cradle rather than the dead end of nephrology, and enthusiasm for the accomplishments of hemodialysis over the years can ultimately outperform frustration over the burden it imposes on patients. Today’s many options to modify hemodialysis for the better represent professional challenges, but these can ultimately be used, right from the start, such as to build the perfect, individualized patient package that should be hemodialysis treatment today.

**Figure:**
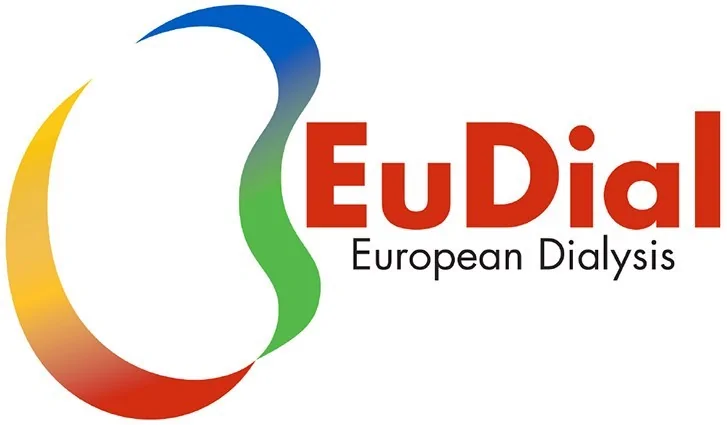


## INTRODUCTION

**Figure 1:**
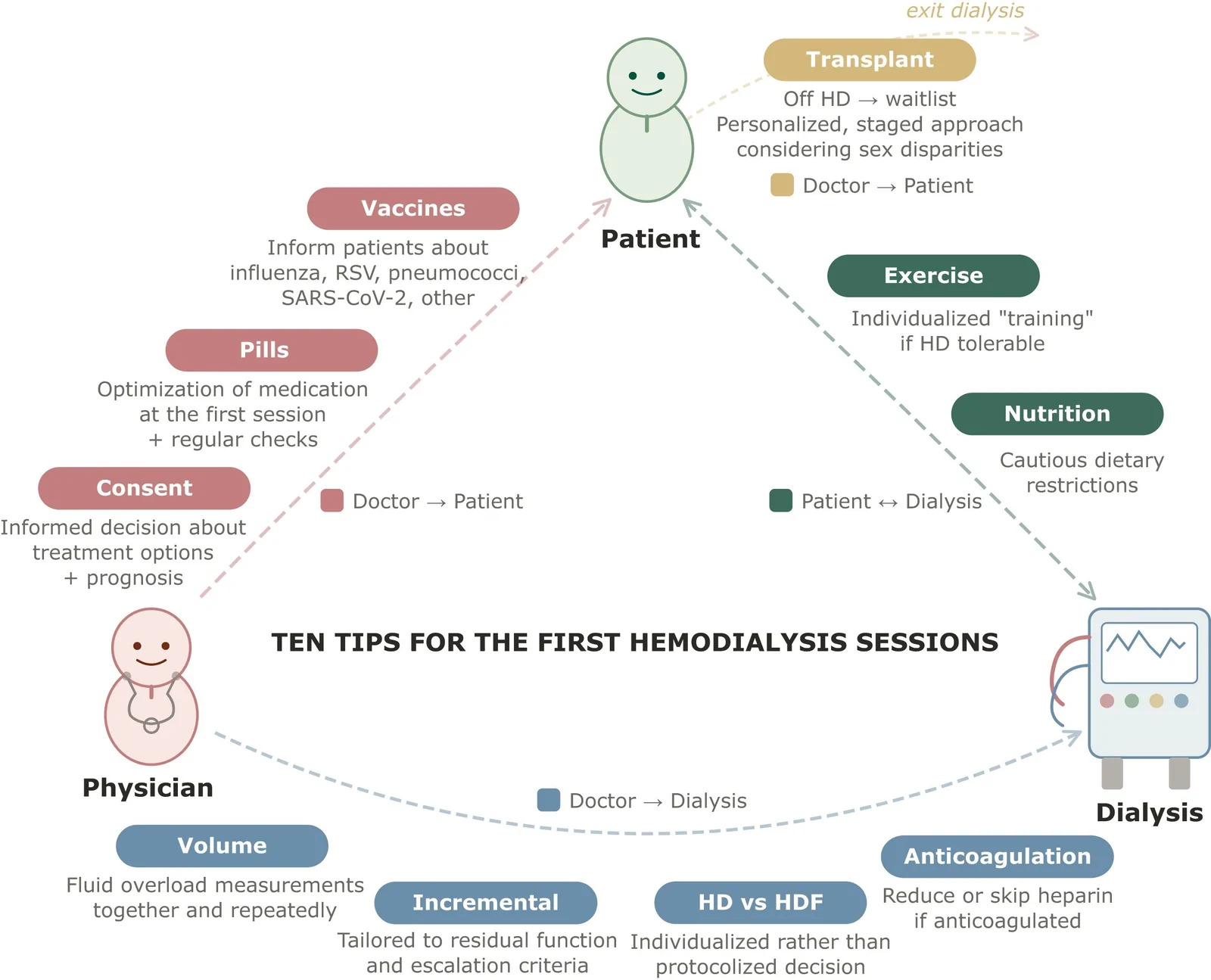
Ten evidence-informed clinical tips for hemodialysis initiation across three pathways. The left arm represents tips requiring shared decision-making between the clinician and patient, prior to or independent of dialysis sessions (Tips 1, 4, and 5). The right arm represents lifestyle tips advised by the clinician but enacted by the patient; the bidirectional arrow reflects the reciprocal influence of the dialysis session on exercise tolerance and nutritional status (Tips 9 and 10). The bottom arc represents purely technical, dialysis-related clinical decisions (tips 2 and 6–8). Transplant eligibility assessment (tip 3) is depicted as an exit pathway (yellow), reflecting the goal of transitioning eligible patients off chronic hemodialysis.

Hemodialysis initiation is perhaps the most consequential turning point in nephrological care, and—as a transition process—was recently reviewed in a comprehensive fashion [1]. Paradoxically, beginning dialysis has been described as representing the actual start of patients’ illness rather than its cure [2], which is an understandable but unfortunate misperception. Here, we provide 10 practical tips for the first hemodialysis sessions (Fig. 1), which intended to support nephrologists of all training stages and members of the multidisciplinary team during such a possibly overwhelming phase. We have been reluctant to provide guideline-like recommendations, which are difficult to accomplish on this topic in an evidence-based fashion. For convenience, reference to the content of previous hemodialysis guidelines is, however, provided in Table 1. Chronic kidney disease (CKD) guidelines on particular topics, if those have also mentioned hemodialysis, are listed in Table 2. The reports from two Kidney Disease Improving Global Outcomes (KDIGO) controversies conferences covering hemodialysis, and two additional consensus documents (on blood pressure in dialysis and hemodialysis versus hemodiafiltration) are summarized in Table 3. Ten tips, altogether, are far too few to cover all of hemodialysis, and important aspects like vascular access [3], anemia management [4], mineral bone disorder (MBD) [5], patient-reported symptoms [6], green hemodialysis [7], and dialysate composition [8] are missing. While the latter issues may require less of a reminder, particularly at hemodialysis initiation, our personal choice can—for didactic reasons—be divided up into items that address patients directly or indirectly, through the dialysis prescription (further explained in the legend for Fig. 1).

**Table 1:** Summary of existing hemodialysis guidelines.

Recommendations	Hemodialysis guidelines	
Domain	KDOQI Clinical Practice Guideline (2015) [198]	Renal Association Clinical Practice Guideline (2019) [152]
1. Dialysis initiation	1.1 Pre-dialysis education1.2 Decision to initiate maintenance dialysis	—
2a. Frequent and long duration HD	2.1 In-center short frequent HD2.2 Informing patients about risks of short frequent HD2.3 Home long HD considerations2.4 Informing patients about risks of home long HD	2.1 Augmented schedules2.2 Incremental schedules2.3 Conservative schedules
2b. HD in special populations	2.5 Pregnancy: Long frequent HD settings	2.4 Pediatric schedules2.5 Schedules during pregnancy
3. Dialysis dose and adequacy	3.1 Dialysis dose target for thrice-weekly HD3.2 HD dose adjustment3.3 Weekly dialysis dose target for non-thrice-weekly HD	*Dialysis dose in thrice-weekly dialysis schedules*
4. Volume, fluid management, and ultrafiltration	4.1 Minimum session duration for thrice-weekly (4.1.1)4.2 Dietary sodium intake and adequate sodium/water removal (4.2.1)	*Fluid in HD (4.1, 4.2)*
5. Dialysate and membranes	5.1 HD membranes for intermittent HD	*Membrane flux and HDFDialysate (5.1–5.4)*
6. Anticoagulation and adverse events	—.	*AnticoagulationAdverse events (7.1–7.3)*
7. Patient experience and self-management	—	*Patient experience (8.1–8.4)*

HD, hemodialysis; HDF, hemodiafiltration; KDOQI, Kidney Disease Outcomes Quality Initiative.

**Table 2:** Aspects of hemodialysis addressed in selected KDIGO/KDOQI guidelines.^a^

Mineral metabolism (CKD-MBD)	Glycemic control	Lipid metabolism	Anemia	Nutrition
KDIGO CKD-MBD (2017) [158]	KDIGO Diabetes Management in CKD (2022) [199]	KDIGO Lipid Management in CKD (2013) [200]	KDIGO Management of Anemia in CKD (2026) [4]	KDOQI Nutrition in CKD (2020) [157]
*Diagnosis of CKD-MBD*3.1 Biochemical abnormalities (3.1.2–3.1.6)3.2 Bone [**3.2.1–3.2.2 (**Table 1), 3.2.3–3.2.5]3.3 Vascular calcification (3.3.1, 3.3.2)*Treatment of CKD-MBD*4.1 Lowering high serum phosphate and maintaining serum calcium (**4.1.1–4.1.6**, 4.1.7, **4.1.8**, 4.1.9)4.2 Abnormal PTH levels (4.2.3, **4.2.4**, 4.2.5)4.3 Bisphosphonates, other osteoporosis medications, and growth hormone (**4.3.3**, 4.3.4)	1.3 SGLT2i (1.3.6)2.1 Glycemic monitoring [**2.1.1 (Figure 11)**; 2.1.2; 2.1.3 (Figure 12)]2.2 Glycemic targets (2.2.1)3.1 Nutrition intake (3.1.2)3.2 Physical activity (3.2.1, 3.2.4)4 Antihyperglycemic therapies in patients with T2D and CKD [4.1, 4.1.3, (Figures 23 and 27); 4.3 (Figure 25)]	1 Lipid status assessment in adults **(1.1, 1.2)**2 Cholesterol-lowering treatment in adults **(Table 4, 2.3.1, 2.3.2)**5 Triglyceride-lowering treatment in adults **(5.1)**	1 Diagnosis and evaluation of anemia in people with CKD (1.2, Figures 5 and 6)2 Use of iron to treat iron deficiency and anemia in people with CKD [**2.1, 2.2 (**Table 3); 2.1 and 2.5]3 Use of ESA, HIF-PHI, and other agents to treat anemia in people with CKD [3.1.1, 3.1.2 (Figure 8), **3.1.1; 3.2.1; 3.3.1;** 3.4.1–3.4.3; 3.5.3 (Table 8); 3.7.1 and 3.7.5]	*Nutritional assessment*1.6 Protein/calorie intake assessment (1.6.1, **1.6.2, 1.6.3**)*Protein and energy intake*3.0 Protein amount (**3.0.3**, 3.0.4)3.1 Energy intake (**3.1.1**)3.2 Protein type (**3.2.1**)*Nutritional supplementation* 4.1 Oral/enteral/IDPN (**4.1.1**, 4.1.2, **4.1.3**)4.3 LC n-3 PUFA (**4.3.1, 4.3.3, 4.3.6**)*Electrolytes*6.3 Phosphorus (**6.3.1**, 6.3.2)6.4 Potassium (6.4.1, **6.4.2**)6.5 Sodium (**6.5.1, 6.5.3**)

^a^Recommendation statements are displayed in bold; remaining entries are practice points. CKD-MBD, chronic kidney disease–mineral and bone disorder; ESA, erythropoiesis-stimulating agents; HIF-PHI, hypoxia-inducible factor–prolyl hydroxylase inhibitors; KDIGO, Kidney Disease Improving Global Outcomes; PTH, parathyroid hormone; T2D, type 2 diabetes; RAS, renin–angiotensin system; SGLT2i, sodium–glucose cotransporter-2 inhibitor.

**Table 3:** Content of selected controversies conferences and consensus statements related to dialysis.

Controversies conferences		Consensus documents	
Dialysis initiation, modality choice, access, and prescription: KDIGO Controversies Conference (2019) [201]	Blood pressure and volume management in dialysis: KDIGO Controversies Conference (2020) [36]	Hypertension in dialysis patients: a consensus document by the EURECA-m (2017) [202]	Hemodiafiltration versus high-flux hemodialysis—a consensus statement from the EuDial Working Group of the ERA (2025) [131]
**Modality selection:** informed patient choice, tailored to individual circumstances; residual kidney function not sole determinant; transition to preferred modality when feasible.**Home dialysis:** Home dialysis underutilized; adoption should be encouraged, barriers addressed, and patient confidence and frailty regularly assessed to prevent early attrition.**Initiation timing**: delay until eGFR <6 ml/min/1.73 m² in older patients without other indications; advance care planning essential; access referral ideally 6 months prior with risk equations complementing clinical judgment; anorexia, nausea, and fatigue should improve within 3 months.**Pregnancy:** consider intensive prescription; target BUN <18 mmol/l; monitor electrolytes, nutrition, anemia**Access:** Individualized decisions and contingency plans; account for patient characteristics, life goals, future treatment trajectory**Adequacy**: Adequacy: beyond Kt/V (residual function, volume, nutrition, CV, symptoms, patient goals), minimum solute targets remain; “adequate” → “goal-directed dialysis” via shared decision-making; incentives and patient-centered outcomes need alignment and validation.	**BP measurement:** Prefer ABPM, home BP or in-office BP over dialysis—unit BP measurements for diagnosis and management.**BP targets:** No dialysis-specific target established; individualized approach recommended, avoiding excessively low BP.Volume management: Volume overload recognized as a major driver of hypertension; emphasis on dry-weight optimization, sodium restriction, and individualized ultrafiltration.**Antihypertensive therapy:** First-line agents generally extrapolated from the non-dialysis population; treatment should consider intradialytic hemodynamics and patient tolerance.**Dialysis prescription:** BP and volume management should be integrated into dialysis prescription design (UF rate, dialysate sodium, treatment time/frequency).**Patient-centered care:** Management should balance cardiovascular protection, symptom burden, quality of life, and patient preferences.	**Diagnosis:** Hypertension should be defined using ambulatory (44-h BP ≥130/80 mmHg) or home BP measurements (average ≥135/85 mmHg over 6 consecutive non-dialysis days/2 weeks).**Management:** Minimization of sodium gain, optimization of volume status, and use of antihypertensive therapy (β-blockers, ACEIs/ARBs, and CCBs as drug classes supported by limited RCT data)	**Convection volume:** High convection volume (>23 l/session) in HDF associated with reduced overall and CV mortality vs. high-flux HD; effect depends on volume and patient health; more likely achieved with AVF than CVC or graft.**All-cause mortality:** Lower all-cause mortality with HDF vs. high-flux HD; effect depends on convection volume and patient health.**CV events:** CV mortality lower with HDF vs. high-flux HD; effect depends on volume and patient health; no difference in sudden cardiac death risk or intradialytic hypotension.**Infections and hospitalization:** Similar hospitalization risk between modalities; HDF may reduce infection-related mortality.**QoL:** HDF may better preserve physical symptoms, cognitive function, and physical activity; sleep quality and uremic pruritus control similar between modalities.**Biochemical outcomes:** Greater clearance of middle molecular weight toxins with HDF; pre-dialysis phosphate lower with HDF; pre-dialysis β2-microglobulin, nutritional/inflammatory markers, and anemia control similar between modalities.**Implications:** HDF as safe as high-flux HD with proper protocols; environmental impact potentially greater but manageable; limited availability may compromise equal access; cost–utility analysis needed.

ACEIs, angiotensin-converting enzyme inhibitors; ARBs, angiotensin-II receptor blockers; AVF, arteriovenous fistula; BP, blood pressure; BUN, blood urea nitrogen; CCBs, calcium channel blockers; CV, cardiovascular; CVC, central venous catheter; eGFR, estimated glomerular filtration rate; EURECA-m, European Renal and Cardiovascular Medicine; HD, hemodialysis; HDF, hemodiafiltration; RCT, randomized controlled trial; QoL, quality of life; UF, ultrafiltration.

## “DISCLAIMER”

Some of our suggestions (especially tip 1, but also tips 3, 4, and 10) are more relevant for patients who have not been regularly followed in a nephrology outpatient clinic before dialysis initiation. However, our suggestions are clearly not intended for an emergency, e.g. acute hemodialysis setting. We also acknowledge that our tips were grounded on clinical experience rather than a systematic literature review, which was out of scope, but would have revealed better distinction between association and causation in the cited studies across tips. To address this shortcoming, we have indicated “observational evidence,” especially when suggesting that such evidence might still motivate a clinical action, despite possible bias structure (confounding by indication, reverse causation, or selection). We hope that many readers, with these limitations in mind, may find our suggestions useful, and we would feel rewarded if applying them into practice might render the patient–caretaker interaction a bit easier, especially at hemodialysis initiation.

### Tip #1: Ask your patients if they know what hemodialysis means and want to stay

Along with severe psychological distress comes a realistic risk of dying, when hemodialysis is first started [9]. Specifically, in a relatively recent German dataset, the average first-year mortality was 33.8%, and highest early after hemodialysis initiation [10], aligning with the observation that cardiovascular (CV) events peak even within the first weeks [11]. A large proportion of dialysis initiators also experience a substantial decline in functioning (regarding their activities of daily living) during the first year [10, 12]. These observations emphasize the need for pre-dialysis education to make informed choices aligned with personal values and preferences. In an ideal situation, a patient with CKD should start hemodialysis after having received realistic information about the impact of hemodialysis on daily life, and ultimately should consent to the decision to start hemodialysis [13]. Many patients, however, feel they are not well informed and/or unprepared for the burden of dialysis initiation [14–17]. Informed decision-making [13] is poorer in the >65 age group, where 40.6% felt that the decision to start dialysis had been made by the doctor [17]. Importantly, the preferences of patients contrast with reality: 90% of patients with CKD in stages 4 and 5 (40.8% pre-dialysis, 50.5% on dialysis, and 8.7% post-transplantation) indicated it is important that they receive detailed information about their medical condition including prognostic information and 80%–85% wanted information about treatment options including withdrawal from dialysis [18]. Unpreparedness for dialysis may contribute to the high proportion of patients that regret having started hemodialysis [18, 19], and perhaps in consequence, withdrawal from dialysis is one of their major causes of death [20–22]. When dialysis care takers first establish their relationship with a new patient, we suggest that asking if the patient feels well enough prepared for hemodialysis not only is non-redundant, but might even help to render the first contact more meaningful. If a patient really does not know why, and what it means to be on hemodialysis, we suggest reassessing the patient’s understanding, when clinically feasible; allow time for insight with involvement of family members and/or caregivers. Where hemodialysis is acutely life-saving, treatment may need to proceed while continuing the informed consent process as thoroughly as possible. Part of this process should include information on peritoneal dialysis and home hemodialysis, but also conservative care.

### Tip #2: Volume first

In a previous review paper, the chief medical officers (CMOs) of 14 of the largest dialysis providers in the United States proposed a “volume first” approach, based on consensus opinion, which was aimed at “improving clinical outcomes among hemodialysis patients” [23]. Their first consensus opinion was that “extracellular fluid status should be a component of sufficient dialysis, such that approaching normalization of extracellular fluid volume should be a primary goal of dialysis care.” While we do not intend to state that ultrafiltration should be *prioritized over* solute clearance during hemodialysis (and here: during the first hemodialysis sessions), we do agree with the CMOs that an appeal for placing volume first is necessary, as solute clearance is less difficult to achieve than euvolemia [23], and we therefore second their plea by ranking volume early on in our present tips’ hierarchy. *Normalization of extracellular fluid volume* was always a primary goal of kidney replacement therapy, but when hemodialysis was first done with Kolff’s rotating drum artificial kidney, fluid was erratically removed by convection [24], and dialysate was hyponatremic, as well as hyperglycemic [25]. Precisely applying ultrafiltration goals today by “targeting” post-hemodialysis weight is noble, as chronic fluid overload associated with mortality [26, 27]. Especially at dialysis initiation, however, we primarily wish to advise hemodialysis team members to accept how difficult it is to decide by how much a new patient may be fluid overloaded [28]. We suggest identifying a potential target weight early on, ideally informed by several objective measures used repeatedly and together [29, 30]. Target or euvolemic weight can vary marginally in both health [31] as well as in hemodialysis [32], and target weight is sometimes grossly misunderstood [33]. Thus we suggest involving patients into ultrafiltration decisions from the first dialysis sessions forward, explaining to them how chronic fluid overload and interdialytic weight gain are not the same [34], and that all aspects of the weighing process (including clothing [35]) matter. Reaching and maintaining euvolemia is a balancing act between fluid accumulation and dialysis complications [36]. As it is not known how well a patient may tolerate the first hemodialysis sessions, our suggestion to immediately think about volume, target weight, and the associated issues is not to be confused with initially being overly ambitious with fluid removal. Strict target weight enforcement and too rapid fluid removal may lead to intradialytic hypotension, which we cover in a simultaneous 10 tips publication of this journal [REF-CKJ-TBD], alongside with ultrafiltration rates (and their association with morbidity and mortality [37–40]). Reaching target weight also demands caring for the preservation of residual urine volume [41, 42] (which may be aided by continuation of loop diuretics; see Tip #4). Diuresis not only impacts fluid management (potentially decreasing interdialytic weight gain) but can also allow for incremental approaches depending on residual urea clearance (see Tip #6). Achieving euvolemia does not necessarily lead to loss of residual diuresis [41], although data from hemodialysis that would be linking objective euvolemia with residual diuresis—to our knowledge—are scarce. In our experience, most patients will agree that euvolemia is the right quest (even from the start of hemodialysis forward). The understandable fear of losing residual urine volume can be overcome by careful monitoring (along with the promise to revert the target weight decision if it was too strict). Recovery time post-dialysis [43] was shown to be associated with quality of life [44] and dialysis treatment characteristics that are relevant to fluid management [45]. Recovery time may be an indicator that dialysis settings are either well tolerated or suboptimal. Monitoring it, hence, allows adding easily interpretable clinical information, which may also help the euvolemia finding process. Returning to hemodialysis development [46], when dialysate sodium >140 mEq/l was found to counteract dialysis disequilibrium [47, 48], the typical 130-mEq/l-concentrations succeeding Kolff were rapidly replaced [24]. However, dialysate sodium concentrations then became lower [49], perhaps due to pathophysiological evidence [50], concern about sodium “loading” [51], and the understanding that sodium reduction led to less thirst and reduced fluid intake [52, 53]. Expert opinions on optimal dialysate sodium are so diverse [54–67] that we suggest remaining tuned in with the literature, which currently indicates that lower dialysate sodium is not better for survival [68] (observational evidence), but worse for intradialytic hypotension and muscle cramps [69]. If dialysate sodium is not individualized to serum, a default setting of 140 mEq/l may be advisable [70] upon dialysis initiation, based on previous [71] and recent [72] (albeit observational) reports.

### Tip #3: Check for transplant eligibility, and then get her on the list

Especially where many patients may feel overwhelmed by dietary restrictions, medications, procedures, and the psychological burden of starting dialysis [73], a brief, but meaningful discussion regarding transplant eligibility may break the ice between a new patient and the hemodialysis team, even during dialysis initiation. The reason is that kidney transplantation is the preferred kidney replacement therapy as it improves survival and quality of life and costs less than dialysis [74, 75], and eligible transplant candidates are likely to appreciate the intention of the hemodialysis team not to withhold transplant listing for the sake of—putting it unorthodoxly—better phosphorous control. This conversation should, however, be preceded by thorough clinical review. The benefits of kidney transplantation extend to older individuals and patients with multiple comorbidities when careful candidate selection and appropriate pre-transplant optimization are applied [76]. Assessing transplant eligibility even before dialysis initiation is a core component of management [76] (see “Disclaimer”), but our advice for the hemodialysis staff is to separate the patient from one’s own, probable frustration if issues have been left unaddressed prior to dialysis start. Longer dialysis vintage before transplantation is associated with higher post-transplant mortality [77, 78], although this association is subject to important confounding, including selection factors and reasons for delayed transplantation. Since candidacy (and potential living donor) work-up often requires months, early identification of potential eligibility may facilitate timely screening, optimization of modifiable risk factors, and avoidance of unnecessary delays. Substantial variation persists in transplant education practices and referral patterns [79], while only 10%–15% of incident patients in a 445-patient cohort had absolute contraindications to transplantation [80]. For example, in a 2019 Canadian cohort, only 1 out of 5 dialysis patients was referred for transplantation within the first dialysis year [81]. This issue is further compounded by persistent sex disparities in access to transplantation, with women (particularly older women) having been shown to be less likely than men to be waitlisted [82–84]. In our opinion, after a meaningful discussion assessing the patient’s wish has taken place, a personalized, staged approach to waitlisting may solve the problem of not overburdening certain patients with additional examinations: Clinicians might proactively continue basic assessments early (especially when overlapping with routine dialysis exams) while avoiding excessive information during the first sessions, if the patient does not wish to engage.

### Tip #4: Pill check: necessary or overkill?

**Figure 2:**
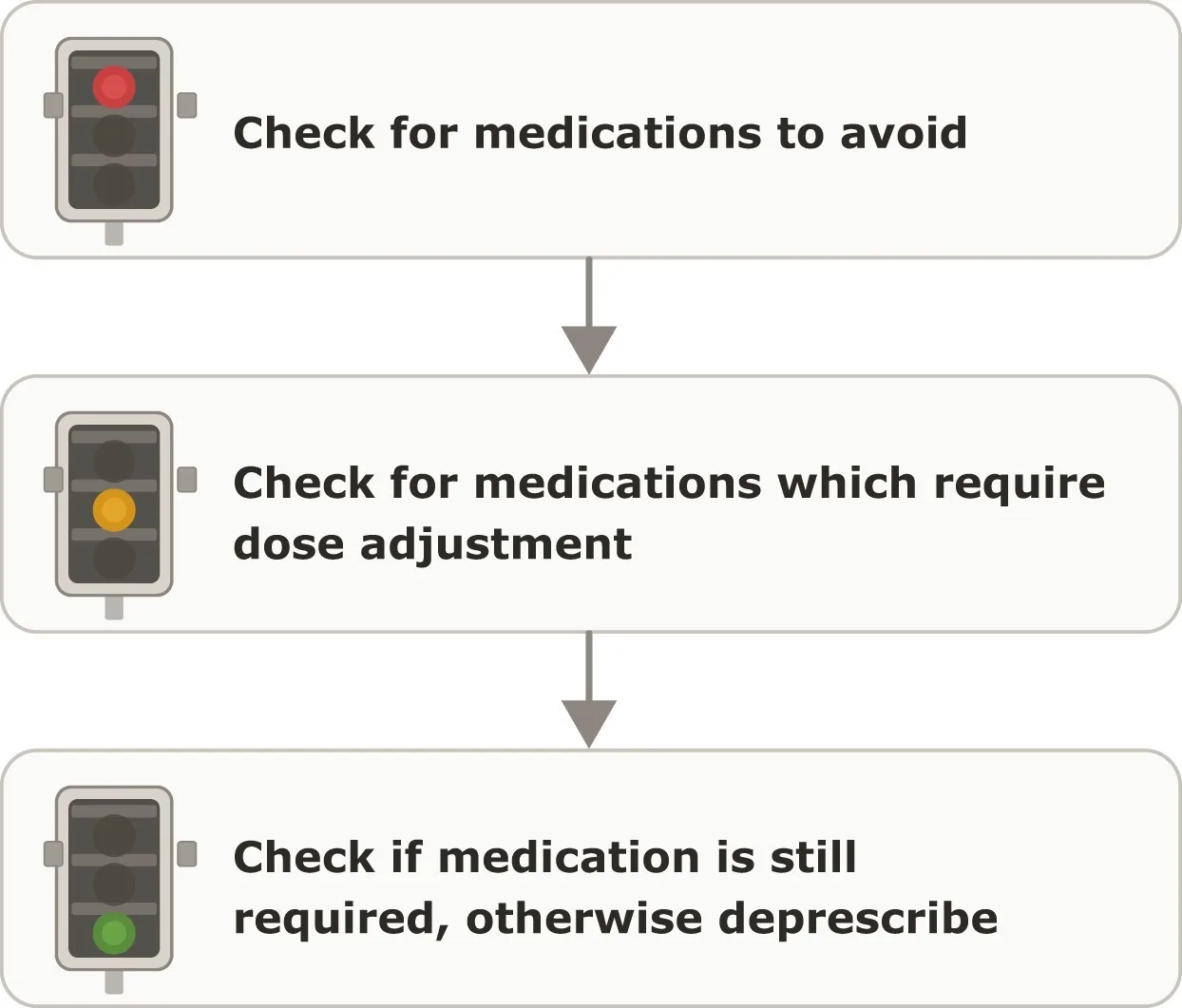
Traffic light system for medication. *Red: Check for medications to avoid*: Some drugs are commonly used in primary care, but should be avoided in advanced CKD [184]; for example non-steroidal anti-inflammatory drugs [185, 186], morphine and codeine [187], baclofen [188], and metformin [189]. *Yellow: Narrow therapeutic index—dose verification and monitoring*. Some drugs have a small therapeutic window in hemodialysis (HD) and require meticulous dose selection and (therapeutic) monitoring to avoid toxicity; for example, gabapentin or pregabalin [190], antihypertensives (check for dialyzability and consider replacement [switching], where relevant [36], antihypertensives may often be unnecessary with active fluid volume management [191], consider overlap with “green” in the suggested traffic light system). *Green: Opportunities to reduce pill burden (“detoxification”)*: Some drugs are rarely discontinued upon dialysis initiation, but have limited evidence for benefit in the HD population and may be de-prescribed safely: oral bicarbonate, phosphate binders [192], and proton pump inhibitors [193]. Regarding diuretics, roughly 20% patients may receive diuretics, even with residual urine volume ≤200 ml [42], which may previously have been considered obsolete (and a welcome opportunity to reduce pill burden). Recent data, however, suggested that loop diuretics may enhance clearance of protein-bound uremic toxins, even in anuria (efficacy and clinical benefits warranting further study [194]). Effects of diuretics on hard outcomes remain inconclusive and evidence is based on observational data: among 11 297 HD patients at a large US dialysis network, those who continued diuretics after dialysis initiation (*n* = 5219) exhibited less frequent hospitalizations, intradialytic hypotension, and a (non-significant) bias toward improved survival compared to controls (*n* = 6078) [195]. These findings were not reproduced in the international Dialysis Outcomes and Practice Patterns Study phases 2–5, where higher doses of loop diuretics (>200 mg/day) were associated with increased hospitalizations in the first year of HD [196]. Several drugs are known to commonly cause weight gain or impede weight loss (as listed in Table 2 in [197])—indications for those could be double-checked, especially in patients who are struggling with obesity prior to waitlisting.

If patients attain their first hemodialysis sessions after having been regularly followed during the pre-dialysis phase, it is important to realize that dialysis is now part of the cure [2] and has the potential to fully or partly correct some of the consequences of CKD, such as metabolic acidosis, hyperkalemia, hyperphosphatemia, and fluid overload-related hypertension, which have previously triggered oral pharmacotherapy. Moreover, iron and erythropoietin-stimulating agents as well as vitamin D can be administered at the end of the session (rather than by patients themselves), and anticoagulation is in many cases debatable (covered in Tip #8). Interestingly, although medication review should already have taken place in those who were followed in the CKD clinic the day before, and are in the dialysis center the day after, patients and doctors sometimes do not seem to look forward to withdrawing potentially unnecessary medication. Perhaps in consequence, polypharmacy, most commonly defined as the simultaneous use of more than five medications [85], is prevalent in hemodialysis patients who—admittedly—often live with multiple chronic conditions, which may render non-prescription a hardship [86]. In Ontario/Canada, for example, more than two-thirds of 3094 patients on chronic hemodialysis were dispensed nine or more different medications [87]. This medication burden increases the likelihood of adverse drug reactions and drug–drug interactions, and is associated with cognitive impairment, greater fall risk, higher risk for hospitalization, and reduced quality of life [85, 88]. To mitigate the “domino effect” of prescribing cascades [89], optimization of the medication list should be started at the first dialysis session of a patient [90], ideally through a structured medication reconciliation process, coupled with a de-prescribing strategy [91]. As a general suggestion, a traffic light-like system may help to provide a standardized approach by, (red), identifying medication with a high potential for harm; (yellow), flagging medications that require intensified monitoring due to narrow therapeutic windows; and (green), highlighting therapies that can safely be discontinued because no longer indicated. Some suggestions for drugs that may be considered “red,” “yellow,” and “green” are provided in the legend for Fig. 2. We also refer to a recent comprehensive review [1], for integration of CKD-MBD and anemia management into the dialysis (therapy) protocol, and for recommendations on whether or not to continue typical heart- and kidney-protective medication [e.g. lipid-lowering drugs, inhibitors of the renin–angiotensin–aldosterone system, non-steroidal mineralocorticoid receptor antagonists, sodium–glucose co-transporter-2 inhibitors, glucagon-like-1 peptide receptor agonists (Table 2 in [1])]. Whatever the value of each drug, dialysis initiation offers a great opportunity to double check its indication, target (outcome), and time horizon for its prescription.

### Tip #5: Shots, shots, shots

In a recent dialysis survey, 86% patients wished to receive the recommended vaccinations, only 39% were aware of the national vaccination plan, and particularly older patients desired more information from their dialysis physician [92]. Response to vaccines is impaired on hemodialysis, related to uremia-associated alterations in innate and adaptive immunity, through oxidative stress, intestinal dysbiosis, and premature immunological aging [93].

#### Influenza

While prior observational studies provided limited certainty on mortality [94, 95], a registry analysis from Taiwan reported fewer cases of respiratory failure among vaccinated hemodialysis patients [96]. Despite lower seroconversion rates in hemodialysis versus general populations [95], high-dose influenza vaccination resulted in more durable seroprotection [97, 98] (while translation into event/mortality reduction is unproven [99]). General population efficacy of mRNA vaccines has to be confirmed in hemodialysis [100]. Timing of vaccination appears to matter, as risk of breakthrough infections increases already 90 days post-vaccination [101]. Data supporting routine use of two influenza vaccinations (“booster”) during a single influenza season are lacking [102, 103].

#### Respiratory syncytial virus

Hospitalizations due to respiratory syncytial virus (RSV) infection are higher among hemodialysis patients than in solid organ transplant recipients [104]. Evidence for RSV vaccination is mainly derived from the general population where hospitalizations and severe disease among vaccinated patients are reduced [105, 106] (observational evidence).

#### Pneumococci

With risk for pneumococcal disease generally augmented in hemodialysis [107], pneumococcal vaccination in this population has been associated with reduced mortality [108, 109]. Patients who never received a pneumococcal vaccine before should receive one Pneumococcal Conjugate Vaccine (PCV) shot, or if previously vaccinated with a Pneumococcal Polysaccharide Vaccine (PPSV23) one PCV booster >1 year after [110].

#### Severe acute respiratory syndrome coronavirus 2 (SARS-CoV-2)

As hemodialysis patients have a higher risk of severe COVID-19 disease, prevention should be prioritized [111, 112]. An extended primary series of vaccination with mRNA vaccines, with subsequent booster doses, might offer sustained protection [112], with both homologous and heterologous vaccine regimens of benefit [113].

#### Other vaccines

High-dose, multiple-dose hepatitis B vaccination regimens, accompanied with post-vaccination serologic testing and timely booster doses, are required to maintain protective immunity [114–116]. While there are limited data on varicella-zoster virus (VZV) vaccine efficacy in hemodialysis patients, they are likely to benefit from VZV vaccination [117].

### Tip #6: Three times a week? Really?

In once or twice-weekly, “incremental” hemodialysis, treatment is tailored to residual urine output, translating into residual kidney function and residual urea clearance [118]. Faster residual kidney function decline in the first dialysis year has been associated with higher all-cause mortality [119], and abrupt initiation of thrice-weekly hemodialysis (standard of care) may accelerate residual kidney function loss during the transition from advanced CKD to intensive dialysis. In an era increasingly focused on individualized medicine, incremental hemodialysis seems like a logical step forward, especially if it is additionally tailored to dietary adherence [120], hypotension, inflammatory status, and hypercatabolism [121]. Nevertheless, evidence so far remains suggestive rather than definitive. Available data are dominated by heterogeneous observational studies, often subject to bias, as patients selected for incremental hemodialysis not only have better residual kidney function, but also fewer comorbidities, and more stable clinical trajectories. Three systematic reviews and meta-analyses reported potential benefits, especially slower residual kidney function decline [122–124]. Randomized data, however, are scarce, and biochemical signals of under-dialysis may occur [125]. Missing the transition point from “appropriate incremental” to “insufficient dialysis” can expose patients to the well-described hazards of long interdialytic intervals. Therefore, a key issue is dose calculation. Kidney Disease Outcomes Quality Initiative (KDOQI) guidelines [126] support estimating total clearance (dialysis + kidney) using residual kidney urea clearance (KRU). Two randomized controlled trials (RCTs) based on the equivalent KRU model [127] are ongoing: IHDIP [128] and REAL LIFE [129]. In short, while we agree we should work toward incremental hemodialysis becoming a new standard of care [130], we also feel obliged to suggest that adequate implementation of this fascinating approach requires a structured approach: residual kidney function reassessment, escalation criteria, and reliable follow-up, shifting complexity from machines to clinicians.

### Tip #7: Hemodialysis or hemodiafiltration: filter freely

The choice between high-flux hemodialysis and online hemodiafiltration in incident patients should be individualized rather than protocolized. RCTs and individual participant data analyses suggest that hemodiafiltration may reduce all-cause and CV mortality, in comparison to hemodialysis. However, these studies are limited by heterogeneous inclusion criteria and methodological shortcomings [131]. Their robustness has been questioned using fragility index and fragility quotient (FQ), which assess how statistically significant results for dichotomous outcomes may be reversed. Although no accepted threshold exists, an FQ ≤ 0.03 should raise concern regarding robustness [132, 133]. In the recent comparison of high-dose hemodiafiltration with high-flux hemodialysis (CONVINCE) trial, reassignment of outcomes in only three participants abolished statistical significance for all-cause mortality (FQ = 0.002) [133]. Similarly, in pooled individual participant data meta-analyses of 4153 patients, overall survival advantage associated with hemodiafiltration disappeared after reassignment of outcomes in only 28 patients [132]. Although survival benefit of hemodiafiltration is clinically relevant, it remains statistically vulnerable. A recent EuDial Working Group consensus statement provided a pragmatic interpretation of evidence for clinical decision-making [131]. Integrating meta-analysis findings, clinical experience, and expert opinion, it identified 22 key points on online hemodiafiltration versus high-flux hemodialysis, particularly for all-cause and CV mortality [131]. Survival benefits appear limited to patients consistently achieving high convection volumes (>23 l/session), typically those with good vascular access and favorable overall clinical status, based primarily on achieved-dose analyses rather than a randomized contrast [131, 132]. During the first hemodialysis sessions, priority should be safety, hemodynamic stability, and progressive adaptation to extracorporeal therapy rather than routine hemodiafiltration prescription (at least in the absence of specific clinical indications favoring hemodiafiltration, such as increased bleeding risk suggesting predilution hemodiafiltration without anticoagulation). Thus, the first hemodialysis sessions may reasonably be performed as standard bicarbonate hemodialysis. Filter size, blood flow rates, and session duration should initially be down-scaled such as to reduce the risk of dialysis disequilibrium syndrome. In parallel, however, the hemodialysis initiation phase can also be used to assess whether high-volume hemodiafiltration is feasible and sustainable through subsequent, stepwise optimization of blood flow, needle size, dialyzer membrane surface area, and treatment time [134–136]. Subsequently, dialysis modality could be changed to hemodiafiltration in selected populations, such as younger patients or transplant candidates with well-functioning vascular access, who may derive greater benefit. Because convection targets may need individualization by patient size, standardization by body surface area may be preferable [137]. After exclusion of dialysis disequilibrium during the very first hemodialysis sessions, high-flux hemodialysis remains the most pragmatic strategy, with transition to hemodiafiltration if high-volume delivery proves feasible and well-tolerated [131].

### Tip #8: Anticoagulated *already*? Maybe skip the heparin

Unfractionated heparin is the most widely used anticoagulant world-wide [138], which prevents clotting (of the extracorporeal circuit) in hemodialysis patients, but must be balanced against elevated bleeding risk [139]. There are no data showing that any type of heparin might be superior to the other, regarding circuit patency, vascular access thrombosis, or major bleeding [140] (meta-analysis of randomized controlled trials and quasi-randomized controlled studies). Where systemic anticoagulation is deemed too risky, saline flushes, heparin-coated membranes, or regional citrate anticoagulation may enable hemodialysis, as per local practice and availability [138]. Particularly during the first dialysis sessions, which are often shorter, systemic anticoagulation may not be required and hemodialysis can frequently be performed without unfractionated or low-molecular-weight heparin. Argatroban, danaparoid, and bivalirudin are viable pharmacological alternatives in heparin-induced thrombocytopenia, again as per local availability. Regional citrate anticoagulation is an alternative strategy [140]. Patients with concomitant anticoagulation for other indications present a specific challenge. Especially prophylactic oral anticoagulation for atrial fibrillation is common and despite extensive discussion in the literature it remains unclear whether and in whom oral anticoagulation should be continued [141] (another 10 tips paper has addressed this topic: [142]). Importantly, traditional risk prediction tools for stroke (e.g. CHA2DS2-VASc) or bleeding risk (e.g. HEMORR(2)HAGES and HAS-BLED) perform poorly and are of very limited use in hemodialysis [143]. A specific dialysis risk score for patients with non-valvular atrial fibrillation has been proposed, but so far has not been studied prospectively [141]. Factors specific to hemodialysis patients, such as future kidney transplantation, may be important aspects when considering whether to initiate, continue, or discontinue oral anticoagulation, as it can complicate unplanned surgical procedures [144]. Whenever oral anticoagulation cannot be discontinued, e.g. in patients with mechanical heart valve or anti-phospholipid antibody syndrome, additional heparin for circuit patency does not have to be given at standard doses. In many cases it can be significantly reduced, thereby decreasing bleeding risk without compromising dialysis quality. Heparin dose should be titrated and adjusted if circuit clotting occurs and dialysis sessions are shortened repeatedly.

### Tip #9: Tell all patients to work out

Individuals receiving hemodialysis are often sedentary, a state consistently associated with increased mortality and poorer health-related quality of life [145–148]. Even modest increases in activity or exercise engagement appear beneficial [149] (observational evidence). However, a distinction must be made between physical activity (everyday movements) and exercise (structured activity to improve fitness) [150]. Exercise can occur independently or during dialysis, aerobic exercise (e.g. [intradialytic] cycling) targeting endurance, resistance training focusing on muscle strength/functional performance [151]. Physicians are central to initiating discussions, ensuring medical appropriateness and identifying contraindications, like volume-related symptoms, recent CV events, or infection [151, 152]. Goals should be defined collaboratively with multidisciplinary teams, including nurses, physiotherapists, and family members, facilitating outside clinic activity [151, 152]. Structured intradialytic exercise is often deferred during the first 3 months [151, 152], a threshold based on expert consensus rather than evidence of harm. The Dialysis Training Therapy multicenter trial randomized patients from 4 weeks onward and demonstrated improvements in physical functioning with 12-month intradialytic exercise [153]. Whether patients in the earliest dialysis period benefit specifically remains unclear, but structured intradialytic exercise should definitely be considered once the adaptation phase has passed [154]. While current CKD guidelines suggest ∼150-min moderate-intensity physical activity weekly [155], this goal is a long-term reference. A pragmatic start involves low-intensity, well-tolerated activity, like 20-min walks on non-dialysis days, with gradual increases [151, 156], early low-threshold physical activity reducing sedentary time. Engagement and progress can be monitored using simple tools (e.g. smartphone step counts or wearable devices) to help maintain motivation [156]. While levels of exercise should be individualized, we suggest that “training” (in the best sense of the term) can be encouraged in all patients who are able to tolerate the hemodialysis session.

### Tip #10: Tell the right patients to eat

Sodium should be restricted to <2.3 g/day (salt <6 g/day) to support volume and blood pressure control [157], phosphorus lowered toward normal, with simultaneous review of calcium and parathyroid hormone (PTH) [157, 158], and dietary potassium adjusted, also toward normal (opinion: [157], refer to [159–162] for food additives and [163] for potassium intake and mortality). If patients receive all of these instructions during the first hemodialysis sessions, some of them may be afraid to eat at all [164]. Insufficient protein and energy intake, however, is highly prevalent and independently associated with mortality, making adequate nourishment an equally important goal as restriction [157, 165, 166]. Recommended targets of 1.0–1.2 g/kg/day of protein and 25–35 kcal/kg/day of energy help prevent muscle loss, functional decline, and fatigue [157], and warrant explicit communication given that patients had previously been instructed to restrict protein intake in advanced CKD (typically 0.55–0.6 g/kg/day) [1]. While obesity in dialysis is burdensome [167] and may require an entirely different approach, especially in patients who will likely be eligible for transplantation [168, 169], we suggest dietary *restrictions* be given cautiously during the first hemodialysis sessions. Among nutritional supplements (complementing dietary management), oral protein-energy supplements are well established [170], but the recent PISCES double-blind RCT [171] moved daily fish oil supplementation (containing omega-3 poly-unsaturated fatty acids [n-3 PUFA]) to the center of attention. In PISCES, the rate of events constituting the primary endpoint (a composite of serious CV events) was 0.31 versus 0.61 events per 1000 patient-days in the fish oil versus placebo group (hazard ratio 0.57, 95% CI 0.47–0.70). At the participant level, at least one event occurred in 20.8% versus 33.7%, an absolute risk reduction of 12.8 percentage points (95% CI 7.9–17.8; number needed to treat 8 over 3.5 years). This effect is directionally consistent with earlier RCTs in dialysis (a significant secondary reduction in myocardial infarction in OPACH [172], improved CV event-free survival in FISH [173]) as well as previous observational data [174–176]. Independent replication is nonetheless awaited, and we therefore wish to present fish oil supplementation as a pragmatic, individualized option rather than a basis for revising guidelines. Biological plausibility supports a beneficial effect, as uremia fundamentally alters high-density lipoprotein composition and function, impairing cholesterol efflux capacity, promoting pro-inflammatory remodeling, and reducing atheroprotection [177–181]. n-3 PUFA may partly restore these defects [182, 183]. A recent survey suggests that patient willingness to supplement is high, though reimbursement was identified as the primary barrier [REF-Niknam-under-review]. While 4-g fish oil (36 kcal) is irrelevant for prevention of protein energy wasting, n-3 PUFA supplementation may be worthwhile discussing with patients and eventually considering at dialysis initiation. Returning to the more conventional nutritional CKD topics (e.g. sodium, phosphorus, potassium), if the dialysis team feels that advice might be useful for a particular hemodialysis initiator, we suggest that dietary counseling and education be *introduced* during the first hemodialysis session but not *delivered* until later, by dietitians or physicians with expertise in nutritional management of hemodialysis patients. This approach, combined with the request for eating (in the “right” patients), we suggest, may enable hemodialysis care takers to address the many aspects already discussed under this tip, and ultimately the other tips presented here.

## Supplementary Material

sfag270_Supplemental_File

## Data Availability

No new data were generated or analyzed in support of this research.
